# Designing a Care Pathway Model – A Case Study of the Outpatient Total Hip Arthroplasty Care Pathway

**DOI:** 10.5334/ijic.2429

**Published:** 2017-03-09

**Authors:** Robin I Oosterholt, Lianne WL Simonse, Stella U Boess, Stephan BW Vehmeijer

**Affiliations:** 1 Delft University of Technology, NL; 2 Reinier de Graaf Hospital, NL

**Keywords:** care pathway model design, integrated care, visual modelling, care model, outpatient total hip arthroplasty, pathway organisation

## Abstract

**Introduction:**

Although the clinical attributes of total hip arthroplasty (THA) care pathways have been thoroughly researched, a detailed understanding of the equally important organisational attributes is still lacking. The aim of this article is to contribute with a model of the outpatient THA care pathway that depicts how the care team should be organised to enable patient discharge on the day of surgery.

**Theory:**

The outpatient THA care pathway enables patients to be discharged on the day of surgery, shortening the length of stay and intensifying the provision and organisation of care. We utilise visual care modelling to construct a visual design of the organisation of the care pathway.

**Methods:**

An embedded case study was conducted of the outpatient THA care pathway at a teaching hospital in the Netherlands. The data were collected using a visual care modelling toolkit in 16 semi-structured interviews. Problems and inefficiencies in the care pathway were identified and addressed in the iterative design process.

**Results:**

The results are two visual models of the most critical phases of the outpatient THA care pathway: diagnosis & preparation (1) and mobilisation & discharge (4). The results show the care team composition, critical value exchanges, and sequence that enable patient discharge on the day of surgery.

**Conclusion:**

The design addressed existing problems and is an optimisation of the case hospital’s pathway. The network of actors consists of the patient (1), radiologist (1), anaesthetist (1), nurse specialist (1), pharmacist (1), orthopaedic surgeon (1,4), physiotherapist (1,4), nurse (4), doctor (4) and patient application (1,4). The critical value exchanges include patient preparation (mental and practical), patient education, aligned care team, efficient sequence of value exchanges, early patient mobilisation, flexible availability of the physiotherapist, functional discharge criteria, joint decision making and availability of the care team.

## Introduction

Nowadays care pathways are widely used in hospitals to manage the decision-making and care processes across medical specialities with the objective to improve the quality of care, increase patient satisfaction, reduce risk and enhance efficiency [1]. New care pathways are developed to improve the organisation of care processes and to further integrate care [2,3]. Fast-track care pathways are a specific type of pathway that aim to give the patient the best available treatment by combining evidence-based clinical features and organisational optimisations, thereby shortening the length of stay (LOS) [4,5,6]. To provide the patient with quality care, clinical optimisations are applied in fast-track total hip arthroplasty (THA) to reduce stress response and organ dysfunction of the patient and shorten the time required for full recovery [5].

Prior research has shown that fast-track THA improves post-operative recovery and reduces the LOS of the patient [7,8,9]. Fast-track THA realisation of higher-quality care has also proven to be more cost-effective [10,11]. Despite the acknowledged impact of the organisational aspects of a care pathway on the LOS [5,6,12,13,14], little research exists on how the care team and care processes should be organised to optimise the LOS in THA care pathways. The clinical results of implemented fast-track THA care pathways are known, but a detailed view on the facilitating organisation, an important part in realising integrated care [2], is still lacking. We undertook the design challenge of bridging this gap with the design of a visual care model [15,16] that supports the adoption of care pathways by providing insight into organisational aspects. Visual modelling is part of the intellectual skill of designers and has been used to some extent used for care modelling in eHealth services [16]. However, more experiments and examples of practice are needed to contribute to the knowledge base and sophistication of the tools of visual care modelling [15].

The aim of this paper is to contribute with a care pathway model of the outpatient THA care pathway that depicts how the care team should be organised to enable patient discharge on the day of surgery. This serves to give orthopaedists and health professionals in the field of THA new insights into the organisational attributes of an outpatient THA care pathway.

In the next section we provide theoretical background on the outpatient THA care pathway, the concept of care model design and how we utilise it in designing a care pathway model. Then we introduce the methodology used, in particular the case study method and the visual modelling toolkit. In the results section we present the outpatient THA care pathway model in two parts and address the optimisations incorporated in each of them. The discussion addresses the contribution and implications of our results and the limitations and suggestions for future research.

## Theoretical background

### Outpatient Total Hip Arthroplasty care pathway

A care pathway is *‘a complex intervention for the mutual decision making and organisation of care processes for a well-defined group of patients during a well-defined period’* [13, p. 137].

The object of this research is the clinical innovation, the outpatient THA care fast-track protocol to be integrated into a pathway design. It concerns a care pathway that enables a patient to be discharged on the day of surgery, and provides the patient with support for returning to their home environment. The LOS is shortened from a typical 4.6 days [9] to a same day discharge. [17,18]. The fast-track protocol used by Hartog et al. [18] is the same for both the overnight and outpatient THA care pathways, but organisational changes are not incorporated in the outpatient pathway yet. The changes increase the intensity of the provision and organisation of care. The result is that the medical treatment can be provided within a shorter timeframe, only one day, with discharge on the day of surgery. Thus far the results are that no outpatient THA complications or readmissions have been reported [17,18], while objective physical benefits have yet to be identified [17]. The hospital organisation benefits from an outpatient THA care pathway as it saves resources and reduces costs [19].

The establishment of a THA care pathway does not ensure these successful outcomes in itself; the effective coordination of the care activities and team are important elements [5,6,12,14]. A THA care pathway is highly dependent on its model of organisation. Improving the organisational flow is considered to be an important future strategy to further optimise the care delivery:

*‘Strategies to improve the organisational flow may be warranted, even mandatory, for further improvement as waiting for physiotherapy, radiographs to be taken, crutches to be handed out, a surgeon to appear for discharge etc. may be barriers for early discharge when the functional discharge criteria are fulfilled’* [6, p. 31].

### Care model design

A model represents a simplified reality, allowing us to manage complexity and to reason accordingly [20]. Models communicate in a visual manner. They are able to transfer and translate knowledge across organisational boundaries [21]. Visual modelling emerged in the design community where designers think and communicate in a visual manner and translate abstract requirements into concrete objects such as 2D and 3D images and physical objects [22]. Models represent reality in a simplified way at different levels of analysis. At a macro level, the chronic care model addresses how chronic care should be organised within a nationwide health system [23]. At a meso level, business model designs describe how organisations create and deliver value in a profit context [24,25,26].

We start from the definition of a business model as defined by Amit & Zott [24, p. 511]:

‘*A business model depicts the content, structure, and governance of transactions designed so as to create value through the exploitation of business opportunities.*’

The content represents the information or goods that are being exchanged and the structure specifies the parties (network of actors) that participate in the exchange and how they are linked. The governance shows the ways in which flows of information, resources and goods are coordinated by the involved parties [24].

This paper presents a micro-level analysis involving the design of a care pathway model in a hospital organisation. To construct this model, we utilise the concept of business model design to capture the value creation and delivery in care model design [15,16].

#### Network of actors

The network of actors is the key element in business model design; it enables the value exchanges and the actors collectively account for the value creation in the model [24]. The network consists of different partners across company boundaries who are linked by value exchanges [27]. The persons representing these partners are the actors in the network. Translated to care pathway model design, we focus on the organisational network of the care pathway, actors connected by value exchanges that span organisational departments. To construct the model we analyse and utilise the actor positions to design the organisational network [16].

#### Value exchanges

**Figure 1:**
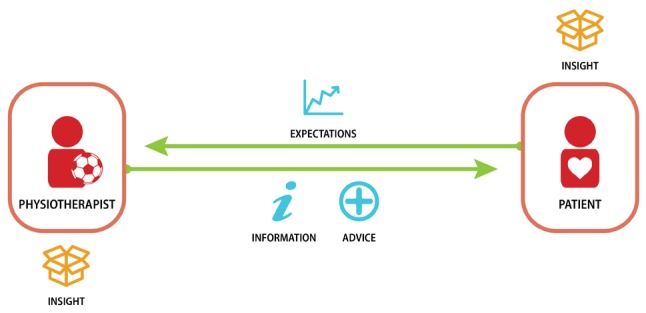
Example of value exchange.

The value exchanges connect the actors within the network. Figure 1 shows an example of a value exchange. Two actors, a patient and a physiotherapist, participate in the exchange. The patient discusses his or her expectations regarding recovery after surgery with the physiotherapist. The physiotherapist informs the patient about the rehabilitation procedure and provides advice on how to prepare adequately. The transaction enables the physiotherapist to understand the patient’s needs in order to provide the right care. The patient gains insight into the rehabilitation procedure and preparation.

The value exchange consists of the transaction content and the value attribute. The content relates to what is transacted in the value exchange, as displayed in the centre of Figure 1. Examples of transaction content and value attributes are shown in Table 1.

**Table 1:** Examples of transaction content and value attributes.

Transaction content		Value attribute	

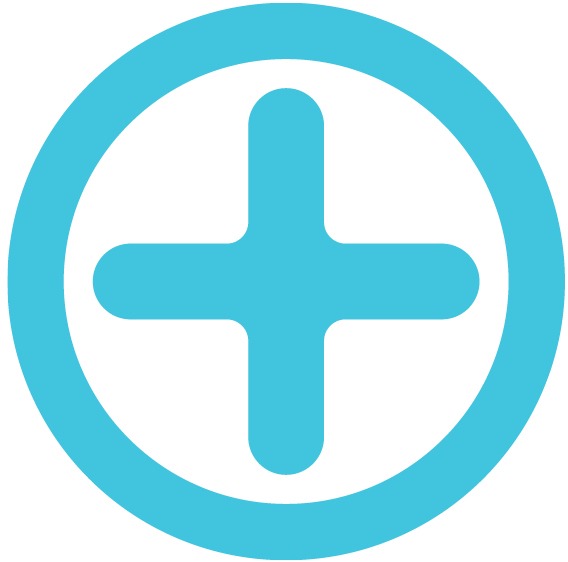	Advice	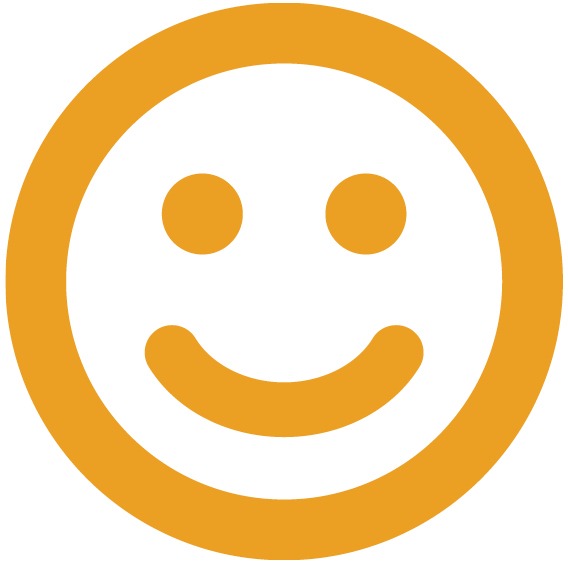	Satisfaction
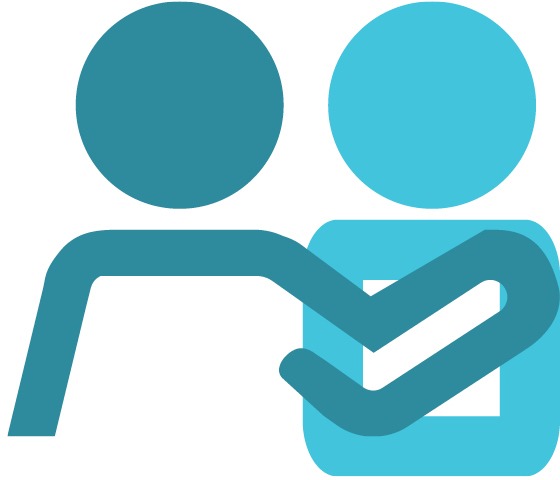	Reassurance	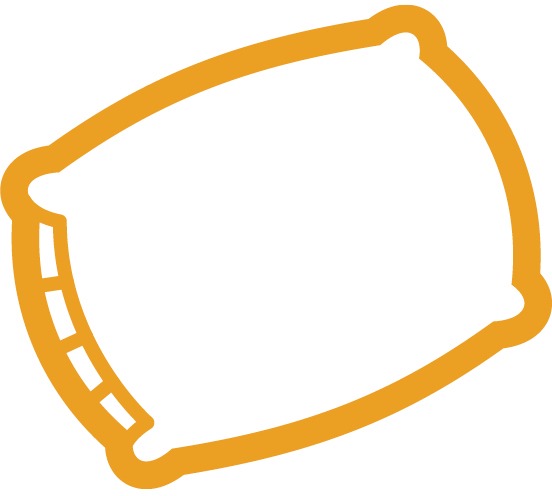	Comfort
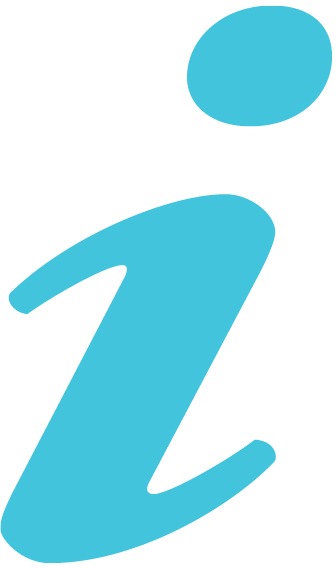	Information	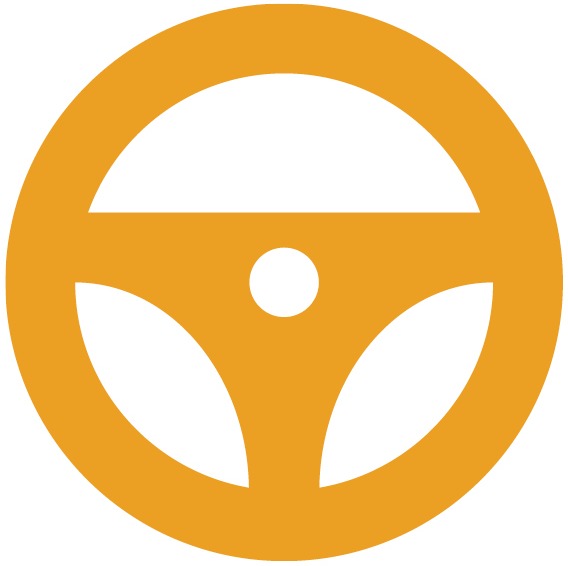	Control (over)
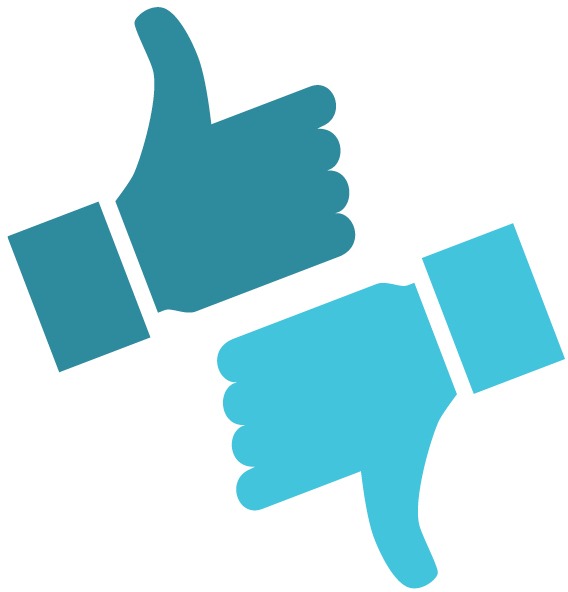	Feedback	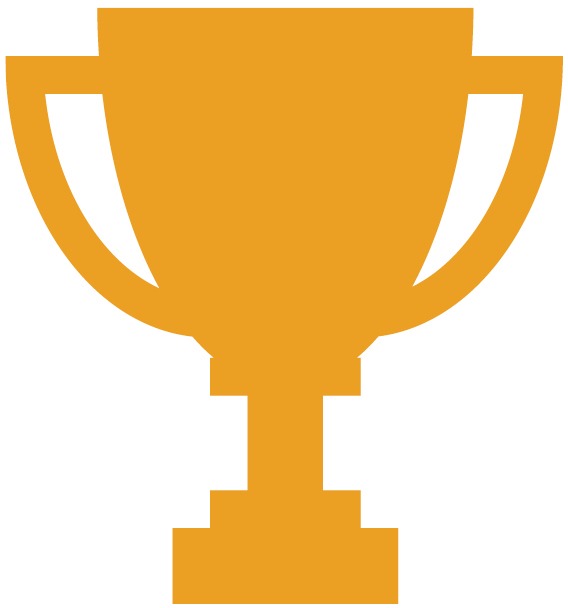	Quality

### Design of care pathway model – research questions

With the design of the outpatient THA care pathway model we add the organisational attributes to the existing clinical attributes in order to support the adoption of the innovation at other hospitals. We postulate a care pathway model as:

*A visual representation of the organisation of the care pathway, depicting the network structure of actors connected by the value exchange*.

We utilise visual care modelling to construct the visual representation. The model defines the roles of the different actors within the network and describes the governance and sequence of the value exchanges. The care pathway model design serves to represent the organisational reality and to translate the embedded knowledge of the outpatient THA care pathway.

Research question

- What is the optimal design of the outpatient THA care pathway model?

Sub-questions

- What value exchange problems and inefficiencies exist within the outpatient THA care pathway at the Reinier de Graaf hospital?
- In the optimal design, what transactions take place in the THA outpatient care pathway model?
- How is the network structured and what are the roles of the actors?
- What is the content and value of the exchanges?

To construct the optimal design of an outpatient THA care pathway model we identify problems and inefficiencies in an existing outpatient THA care pathway. We then design solutions that solve these specific problems and inefficiencies. The optimal design of the outpatient THA care pathway model depicts the network structure of actors connected by value exchanges. It defines the roles of the actors and the content of the value exchanges.

## Method

### Case study

The design of the care pathway model requires a detailed understanding of the outpatient THA care pathway. A single embedded case study research method is applied to gain insight into the organisation of a complex care system of the pathway and its hospital context [28], while retaining its holistic and meaningful characteristics [29].In this case study research we build theory from in-depth case data analysis [30]. While coding is the common approach for qualitative data analysis [30,31], we employ a tailored visual mapping tool. Such tools are

‘*particularly attractive for the analysis of process data because they allow the simultaneous representation of a large number of dimensions, and they can easily be used to show precedence, parallel processes, and the passage of time*’ [32, p. 700].

The visual representation of the care pathway model is an abstract conceptualisation, an intermediary step to construct theory [32].

The Reinier de Graaf hospital was selected for our in-depth case study. At the time of the research the Reinier de Graaf hospital was the only hospital in the Netherlands with a fast-track outpatient THA care pathway and the first European healthcare organisation to publish about such a care pathway [18]. Due to its position and deviation from routine THA care pathways, it is an extreme case to study [29,33]. The case concerns a large teaching hospital where seven orthopaedists operate; one of them performs the surgeries in the outpatient THA care pathway. Its key clinical attributes here is a radically reduced length of hospital stay, from 4.6 days to 1 day after the introduction of a rapid recovery protocol for primary THA procedures. This was researched by a cohort study with 1,180 unselected patients and in an outpatient setting with 27 selected patients. Both clinical studies are published in Acta orthopaedica, (2013; 2015) [9,18]. During the research period, between 1 April 2014 and 30 October 2015, 100 patients were discharged in an outpatient setting. The researcher gathered data over a period of four months from January to May 2015 while stationed at the hospital. During this time, 36 patients were discharged in an outpatient setting. This is an embedded case study as the different care processes and employees within the care pathway are studied [29]. The selected case comprises the pre-, peri- and post-operative care period. The object of analysis is the network of actors of the care pathway, with a focus on how the care delivery is organised and how the actors are linked. The orthopaedic surgeon and physician assistant were involved in the research project as they were both highly knowledgeable about the organisation of the care pathway, which enabled us to create a more accurate model. The use of multiple sources limits bias. To support the later transferability of our results we provide a thick description of our case and research [33,34].

The case is representative for outpatient THA care pathways with no overnight stay. The representativeness applies to both regular and teaching hospitals, since they have a similar organisation structure and type of caregivers. The difference between these hospitals is that regular hospitals in the Netherlands do not have caregivers in training, resulting in less overlap and a clearer division in roles. The case is not representative for academic hospitals as they focus on tertiary care, research and education that require a different organisational structure and employees. Moreover, only few THA procedures take place at academic hospitals in the Netherlands.

### Data collection

Semi-structured interviews were conducted to map the transactions and identify problems and inefficiencies within the care pathway. An interview guide was constructed to ensure that the sub-research questions were addressed while still being able to pursue topics of interest that emerge in the interview [35]. As standard interviewing techniques cannot capture a detailed image of the transactions in a complex network structure, a visual care modelling toolkit was developed and applied to map the transactions in the interview. Together with the interview guide the toolkit structured the story of the interviewee and created consistency in the data collection across interviews. A pilot interview was conducted with a participant who had no prior experience with interview toolkits. Minor adjustments were made to improve the method and toolkit.

#### Sample

The interviewees were sampled purposefully based on their involvement in the development and the current operation of the outpatient THA care pathway (Table 2). A total of 11 healthcare professionals within the outpatient THA care pathway were interviewed. Eight of them were also involved in the prior implementation and development of the care pathway. Seven of these professionals are continuing to take part in the development of the care pathway. Additionally, two outpatient THA patients were selected who were in different stadia of post-operation recovery. One patient was in the first week, the other in the seventh. Furthermore, among the actors in the pathway, a representative from a health insurer and two representatives of Biomet were selected. Biomet is a musculoskeletal implant manufacturer and provides the service of care pathway optimisation. The Reinier de Graaf hospital utilises both the implants and the service.

**Table 2:** Sample of interviewees.

Actor	Function	Involved in the pathway’s		
		
		early development	implementation	continuous development^(1)^

**Operational process**				
Patient A	Subject of the outpatient THA care pathway.	×	×	×
Patient B	Subject of the outpatient THA care pathway.	×	×	×
Anaesthetist	Examines the patient to prescribe the right anaesthesia.	√	√	√
Nurse	Ensures the health and safety of patients at the ward.	×	×	×
O.R. Nurse	Assists the orthopaedic surgeon during surgery.	×	×	×
Orthopaedic consultant	Informs and educates patient about medical procedures, and ensures that the right arrangements are made at their homes.	√	√	√
Orthopaedic surgeon	Performs the outpatient THA surgeries and discharges the patient. Introduced the caregivers and hospital to the idea of fast-track surgery and outpatient THA; had a crucial role in ensuring the acceptance and implementation of the care pathway.	√	√	√
Physician assistant	A specialist nurse who is able to execute certain tasks of the orthopaedic surgeon at the outpatient clinic, such as post-operative checks. The chairperson of the different meetings. Has a central role in managing and disseminating the developments in the care pathway. Is often consulted for information by other actors in the care pathway.	√	√	√
Physiotherapist	Assists in the postoperative recovery process of patients.	√	√	√
Ward doctor	An orthopaedist in training who diagnoses patients at the outpatient clinic and visits patients at the ward.	×	×	×
**Managerial process**				
Care manager	Manager of various business units including orthopaedics.	√	√	√
Head of nursing	Manages the orthopaedic nursing department on a day-to-day basis.	√	√	√
Manager orthopaedics	Manages the orthopaedic partnership on a day-to-day basis.	√	√	√
**External process**				
Biomet consultant	Consults healthcare organisations on optimising their care pathways.	×	×	√
Biomet sales representative	Assists healthcare organisations with the use of products and sells products.	×	×	√
Health insurer	In charge of selecting care for their insured.	×	×	×
(1)	Every four to six months, meetings are held with the different caregivers involved in the pathway. Problems are discussed, projects are initiated and progress is tracked on ongoing projects.			

All 16 interviews were conducted in a one-on-one setting by the same interviewer. Due to the central role of value exchanges in the interview, a definition and a visual example (Figure 1) were provided to establish a common understanding. All interviews were audio-recorded, and all mapped value exchanges were photographed after the interview. The orthopaedic surgeon obtained consent from both interviewed patients. The interviews were conducted in Dutch, the native language of all interviewees, allowing the respondents to fully express themselves and enable the interviewer to interpret the responses with a cultural understanding [36]. The health insurer was interviewed via telephone. The visual care modelling toolkit was therefore not used in the interview.

#### Visual care modelling toolkit

The visual care modelling toolkit developed for this research is a modification of the toolkit by Arts-Posthoorn & Gedde [37], which in turn was inspired by the Net-Map tool [38] and the visual brainstorm method by the Board of Innovation. This type of toolkit has earlier been utilised for the care model design of a pre-care e-health service [16], but never before in a care pathway in hospitals.

The toolkit consists of a map and cards. The toolkit map is a large sheet on which the value exchanges, wherein the interviewee participates, are physically mapped. The horizontal axis depicts the different phases that exist in the care pathway (pre-operative, peri-operative, post-operative and others). The vertical axis is left blank for the interviewee to position only the actors that participate in the specific exchange. Where the two axes cross, value exchanges are mapped using toolkit cards. Three categories of cards were prepared and presented in the interviews: actors, transaction content and value attribute cards, each in a distinctive colour. Each card contained an icon and a representative word (Table 1). The cards were pre-printed and presented at the interviews. Blank cards provided the interviewee with the flexibility to add missing cards and adequately describe actors or value exchanges.

### Data analysis

#### Constructing the network of actors

Three types of qualitative data were analysed: audio recordings, maps and documentation. After each interview the photographed map was digitally reconstructed in Illustrator (CS6 v16) to facilitate comparison and analysis by establishing order and clarity. The 15 digital maps were comparatively analysed at a network level to determine which actors participate together in value exchanges. Due to an excessive amount of different connections per actor, the network was split into phases for the sake of comprehensibility. After several iterations a clear view on the composition of the organisational network was established. Five successive phases of the care pathway were constructed and digitally visualised: diagnosis & preparation (1), admission (2), surgery (3), mobilisation & discharge (4), home recovery & checks (5).

#### Identifying and mapping value exchanges

After reconstructing the network structure of the care pathway, the content and value of the value exchanges were determined. For this purpose the audio recordings were partially transcribed with InqScribe (v2.2). The first step in mapping a value exchange was to analyse the maps of the actors that participate in the exchange. This served to get an initial understanding of the transaction and to spot commonalities and differences. Next, the audio recordings of the exchanges between the actors were analysed in a similar fashion, cross-checking the value exchange between two sources. Once the value exchange was extracted from the data it was mapped. If the transaction remained unclear or could only be analysed unilaterally, an additional source was consulted to verify the transaction. The value exchanges were added to the network structure of each phase, constructing the model of the outpatient THA care pathway in its current state.

#### Selecting organisational problems and inefficiencies

The interviewees mentioned various problems or inefficiencies in the care pathway. They were all listed and sorted on the basis of shared themes to identify commonalities and dependencies. The problems and inefficiencies were analysed to determine whether they are clearly part of the value exchanges or organisational network. Problems that could not be addressed in the model were left out of scope, such as outdated patient information (Table 3).

**Table 3:** Organisation problems and inefficiencies.

Care pathway	

Poor communication on the implementation of changes in the outpatient THA care pathway.	√
Lack of information-sharing and transparency between caregivers causing problems in the provision of care.	√
The ward doctor alternates monthly between specialists in training, which requires time to adjust and settle in the role of preparing, examining and prescribing the right medications to the patient.	
The different actors of the care pathway do not feel responsible for the pathway’s development, as a result of which problems are neglected, making it difficult to improve the care pathway.	√
**Model phase 1**	

The information provided to the patient (education) is outdated, repetitive, excessive and generic.	
Inefficient consultations with the orthopaedic consultant, long and redundant.	√
Patient does not always prepare for the orthopaedic consultation by reading the information folder.	
Many patients do not visit the group information session pre-operatively.	√
High number of pre-operative patient transactions and hospital visits, which is very time-consuming, especially for patients traveling from afar.	√
The anaesthetist has not always enlisted the medication in the patient’s file at the time the patient visits the ward doctor pre-operatively; this results in that the ward doctor is unable to provide a patient prescription, causing issues at a later time.	√
**Model phase 4**	

The recovery time from spinal anaesthesia varies per patient, making it difficult to predict when mobilisation can take place.	
Misunderstanding between caregivers on the patient treatment. The agreement is that only the first two patients may be discharged on the day of surgery. Even if the third patient meets the discharge criteria he or she cannot go home.	
The ward doctor’s presence is needed at both the ward and outpatient clinic. However in the afternoon the ward doctor is at the outpatient clinic and rarely visits the ward, which means the patients only get to see a doctor at discharge.	√

#### Outpatient THA care pathway model design

The various problems and inefficiencies formed the input for the design of the outpatient THA care pathway model. The design process is iterative and consists, broadly, of analysis, synthesis and evaluation. Each problem was addressed separately, starting with an analysis to establish a thorough understanding of the problem and its context. In some cases actors were reconsulted to gain this understanding. The analysis was followed by a creative phase seeking to tackle the problem by combining creative thinking and idea generation to generate solutions. The solutions were then evaluated on how well the problem was solved and how this affects the different stakeholders. These different phases were iterative and often progressed in parallel, with the problem and solution emerging together.

## Results

### Organisation problems and inefficiencies in the pathway

Table 3 shows the different problems in the outpatient THA care pathway at the case hospital. The overall organisational problems that span all phases in the care pathway are: a lack of information-sharing and transparency between caregivers, poor communication on the implementation of changes and a lack of responsibility for the pathway’s development.

The two most critical phases of the outpatient THA care pathway are the *diagnosis & preparation* (1) and *mobilisation & discharge* (4) phases. These phases are crucial to enable same-day discharge from an organisational perspective because in these two phases the organisational interactions transform the most due to new type of expectation management concerning fast-track recovery of the patient. The problems listed in Table 3 concerning these phases are addressed in the paragraphs presenting the corresponding model phase. The other phases (admission, surgery and home recovery & checks) are not presented in this paper, firstly because they are not crucial, at an organisational level, for enabling discharge on the day of surgery, and secondly because they are generic and similar across hospitals. They have been modelled in the same manner. The problems followed by a checkmark in Table 3 are addressed in the presented design.

### Model phase 1 – diagnosis and preparation

**Figure 2:**
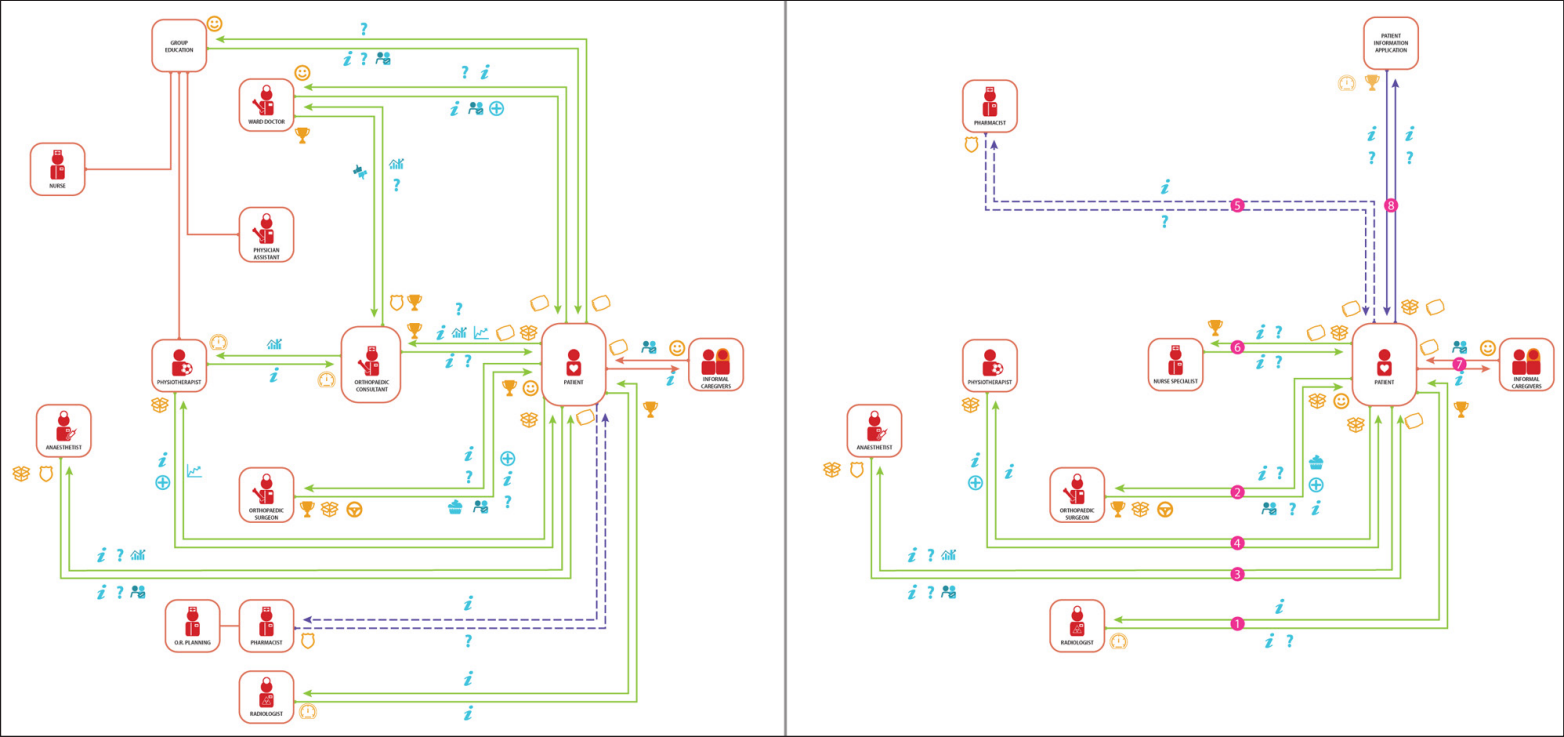
Model phase 1 diagnosis and preparation, as-is (left), optimised design (right).

The pathway model design of the first care phase visualises the network of actors and value exchanges for the diagnosis and preparation of the patient. Figure 2 shows the as-is care pathway at the case hospital and the optimised design side by side. The value exchanges in the design are listed in sequence (Table 4) and the role characteristics of the actors are stated in the appendix. The design removes problems and inefficiencies. It involves nine actors (reduction of two) and a total of eight patient-involved value exchanges (reduction of two). The patient visits the hospital on two separate occasions (reduction of two). The critical organisational attributes of this phase are patient preparation (mental and practical), patient education, aligned care team and efficient sequence of value exchanges.

**Table 4:** Value exchanges of care pathway model design phase 1.

No.	Involved actors	Transaction content	Value attribute

**Patient visit 1**			
1	Radiologist, Patient	The radiologist takes an x-ray of the patient’s hip and provides the patient with information about the procedure. The patient answers any clarifying questions from the radiologist.	Patient: quality Radiologist: efficiency
2	Orthopaedic surgeon, Patient	The orthopaedic surgeon examines and questions the patient for diagnosis. The patient is provided with information about his or her condition, treatment options are advised and discussed, and the patient is reassured. The patient asks any questions he or she has and both actors exchange and discuss their expectations concerning the procedure and the post-operative recovery. Finally the orthopaedic surgeon conducts a physical examination and motivates the patient to recover.	Orthopaedic surgeon: insight, control, quality Patient: insight, satisfaction
**Patient visit 2**			
3	Anaesthetist, Patient	The anaesthetist questions the patient about his or her health and gathers medical data (e.g. drug use, blood pressure). The patient is reassured and discusses the anaesthetic procedure with the anaesthetist. The patient answers and asks questions.	Anaesthetist: insight, safety Patient: comfort
4	Physiotherapist, Patient	During the physiotherapeutic consultation the recovery process and expectations of the patient are discussed. The physiotherapist provides the patient with information and advice. During the consultation the patient is taught to walk with crutches.	Physiotherapist: insight Patient: insight
5	Pharmacist, Patient	The pharmacist phones (dotted line) the patient to check which drugs the patient uses. The patient provides the pharmacist with the needed information.	Pharmacist: safety Patient: comfort
6	Nurse specialist, Patient	The nurse specialist conducts the medical anamnesis by questioning the patient and ensures that the appropriate arrangements are made at home to support discharge on the day of surgery. These are, for example, the availability of an informal caregiver for support on the first day and moving the bed to avoid excessive use of stairs. The nurse specialist prescribes the medication needed after discharge so that it is already available when the patient returns home. The patient provides the needed information and asks questions to remove any uncertainties.	Nurse specialist: quality Patient: comfort, insight
7	Informal caregiver, Patient	*continuous* The informal caregivers are (generally) present at all value exchanges involving the patient. They reassure the patient, while the patient provides information to the informal caregivers about the care pathway.	Informal caregiver: satisfaction Patient: comfort
8	Patient information application, Patient	*continuous* The patient information application informs the patient throughout the entire pathway about his or her journey, preparing the patient on what to expect and when. The patient is able to ask urgent questions via the application. The date of surgery is scheduled via this application.	Patient information application: quality, efficiency Patient: insight, comfort

Mental preparation of the patient is essential for a discharge on the day of surgery. The patient is educated about his or her hospital journey to manage their expectations on the different care processes and mentally prepare them for discharge on the day of surgery. Any doubt in the patient’s mind will hinder discharge. It is imperative that all caregivers involved in the care pathway are aligned and agree on the treatment for these patient groups. Any disagreement between caregivers will affect the patient and have adverse effects. The practical preparations to support the patient in discharge on the day of surgery include practicing walking with crutches, prescribing post-discharge home medication and assessing the patient’s home situation to ensure the right arrangements are in place to support the patient on the first few days at home. Due to the caregivers’ interdependency, the sequence of the value exchanges is important for the efficiency and continuity of care. For example, the nurse specialist cannot provide a medical prescription if the patient has not been examined by the anaesthetist.

#### Model phase 1 – designed optimisations for diagnosis and preparation

The new model design improves (1) the education of the patient, (2) the process quality of diagnoses and preparation and (3) the efficiency of care. (1) In the analysed situation (as-is) patients are educated by an orthopaedic consultant on two separate occasions and during a group session. Patients and caregivers alike experience these educative transactions as lengthy and repetitive, consuming valuable resources. The redesigned model involves the support of a patient information application to educate and manage the expectations of the patient.

(2) Group education and orthopaedic consultations have both been replaced. The nurse specialist replaces the orthopaedic consultant by conducting the medical anamnesis. The digital patient information application provides the patient education: It gives the patient the right information at the right time and schedules the date of the surgery. With this, the patient is optimally prepared for the outpatient THA care pathway.

(3) The efficiency optimisation is the replacement of the outpatient ward doctor who is currently (as-is) an orthopaedist in training at the case hospital. The ward doctor’s presence is required at both the ward and outpatient clinic. To create a more generic and comprehensible model the responsibilities formerly fulfilled by the ward doctor are in the hands of the orthopaedic surgeon (conducting the physical diagnosis), the nurse specialist (prescribing the right medication) and the nurse (marking of patient’s leg on the day of surgery; phase 2).

### Model phase 4 – mobilisation and discharge

**Figure 3:**
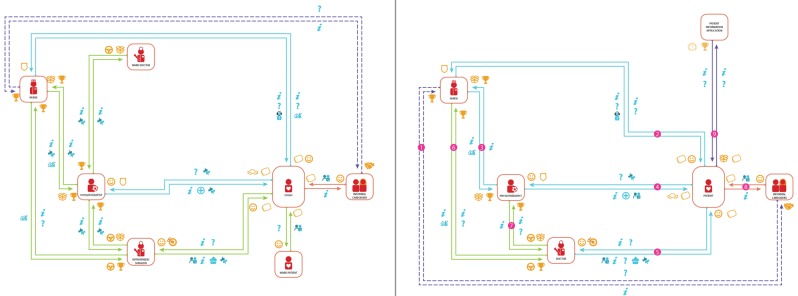
Model phase 4 mobilisation and discharge, as-is (left), optimised design (right).

The pathway model design of the fourth care phase visualises the network of actors and value exchanges for the recovery and discharge of the patient at the ward. Figure 3 shows the as-is care pathway at the case hospital and the optimised design side by side. The value exchanges in the design are listed in Table 5 and the role characteristics of the actors are stated in the appendix. The design removes problems and inefficiencies. It involves six actors (reduction of one) and a total of five patient-involved value exchanges (unchanged from as-is).

**Table 5:** Value exchanges of care pathway model design phase 4.

No.	Involved actors	Transaction content	Value attribute

1	Nurse, Informal caregiver	The nurse phones the informal caregivers to inform them that the patient has returned to the ward. Any questions the informal caregivers have are answered.	Nurse: quality Informal caregiver: reliability
The following transactions are not listed in a strict successive order, but strong dependencies exist.			
2	Nurse, Patient	*continuous* The nurse is the main contact person for the patient and supports him or her at the ward by checking in regularly, asking questions and monitoring the patient's health. In turn the patient provides information to the nurse and both asks and answers questions. This transaction is dynamic and occurs multiple times (blue colour). The nurse handles the discharge of the patient, informs him or her about do's and don'ts and arranges all the needed paperwork at discharge.	Nurse: safety Patient: comfort
3	Nurse, Physiotherapist	The nurse informs the physiotherapist when the patient has returned to the ward. Relevant medical patient data and information about how the patient feels is exchanged to determine when mobilisation of the patient could take place. At a later point in time the physiotherapist informs the nurse regarding progress in mobilisation. Both actors inform each other to track progress and coordinate whether the patient can be safely discharged.	Nurse: quality Physiotherapist: quality, insight
4	Physiotherapist, Patient	(*twice*) The physiotherapist supports the patient’s mobilisation on two separate occasions, providing advice and reassurance. The patient asks questions and gives feedback on mobilisation. The patient uses a walker for support during the first mobilisation. During the second, the patient uses crutches to meet the functional discharge criteria.	Physiotherapist: satisfaction, safety Patient: mobility, comfort
5	Doctor, Patient	The doctor visits the patient twice after surgery to check progress. The doctor explains how the surgery went and asks the patient how he or she is doing. The patient provides information on how he or she feels and asks any questions he or she still might have. Lastly the doctor reassures and motivates the patient. If the patient meets the functional discharge criteria and both the physiotherapist and nurse agree, the patient is discharged.	Pharmacist: safety Patient: comfort
6	Doctor, Nurse	The doctor asks the nurse how the patient is doing and whether the patient is able to go home. The nurse bases the response on medical data and information about the patient. Finally, the doctor notifies the nurse when patient discharge is approved.	Nurse specialist: quality Patient: comfort, insight
7	Doctor, Physiotherapist	The physiotherapist exchanges information with the doctor, and the doctor asks questions to discuss the state of health of the patient in order to determine whether it is safe to discharge the patient.	Informal caregiver: satisfaction Patient: comfort
8	Informal caregiver, Patient	*continuous* The informal caregivers are (generally) present at all value exchanges involving patients. They reassure the patient, while the patient provides information to the informal caregivers about the care pathway.	Informal caregiver: satisfaction Patient: comfort
9	Patient information application, Patient	continuous The patient information application informs the patient throughout the entire pathway about his or her journey, preparing the patient for what to expect and when. The patient can ask urgent questions via the application. The date of surgery is scheduled via this application.	Patient information application: quality, efficiency Patient: insight, comfort

The critical organisational attributes of this phase are early patient mobilisation, flexible availability of the physiotherapist, functional discharge criteria, joint decision making and availability of the care team. The most important aspect in enabling fast recovery in this phase is the mobilisation of the patient a few hours after surgery. The physiotherapist mobilises the patient on two separate occasions, early and late afternoon. Pre-operative anaesthesia directly influences the time of mobilisation as dosage and effect varies per patient. The physiotherapist therefore checks the patient at the ward to see whether the anaesthesia has worn off; if it has, he or she mobilises the patient. For this purpose, the physiotherapist must have a flexible schedule. For the second mobilisation the patient has to meet the functional discharge criteria: walk 30 m, climb stairs, dress independently and independently visit the toilet. The nurse ensures the safety of the patient in this phase and closely collaborates with the physiotherapist to discuss the patient’s recovery progress and mobilisation schedule. The nurse, physiotherapist and doctor jointly reach consensus on the patient discharge. In addition to the functional discharge criteria the wound has to be dry and patients should not be dizzy or nauseous. The final decision to go home is up to the patient. The goal is to provide the patient with the best care available. In the event that a patient does not feel it is safe to go home even when the discharge criteria are met, an overnight stay is fully supported.

There is flexibility in the sequence of the value exchanges, but there are strong dependencies. The doctor cannot discharge the patient without prior mobilisation by the physiotherapist. The time of discharge is dependent on the work hours of the care team. At the case hospital, approval for discharge is commonly given at 18:00 p.m. Discharges at a later time do happen, for example due to a high dose of anaesthesia or a delay in the operating room. To support patient discharge after standard work hours, the relevant caregivers (doctor, physiotherapist and nurse) have to have clear agreements about permission to discharge after a certain time and its influence on the availability of the caregivers.

#### Model phase 4 – designed optimisations for mobilisation and discharge

This new model design improves (1) the communication interactions with the patient, (2) the process quality of diagnoses and preparation and (3) the efficiency of care.

(1) It provides the patient with more frequent quality contact during a short stay at the hospital. Furthermore, thanks to the addition of the patient information application, the patient receives the right information at the right time.

(2 & 3) In the analysed situation (as-is) the ward doctor rarely visits the patients at the ward post-operatively because he or she has to be at the outpatient clinic in the afternoon. As a doctor’s visit is beneficial in tracking the recovery progress of the patient, the ward doctor and orthopaedic surgeon are merged into the role of doctor. This role can be shared by multiple actors (if needed) to ensure the patient is visited multiple times by a doctor: at discharge and once or more prior to it. This provides the doctor with a better view of how the patient is progressing.

### Similarities and differences

Three organisational problems spanning the entire pathway were addressed by introducing a digital caregiver platform in combination with a patient feedback application. This caregiver platform enables all team members to: identify problems in the pathway based on the visualised satisfaction ratings of the patients, initiate projects to solve problems, and collaborate in a digital environment unrestricted by time.

The two optimised models differ in the purpose of the value exchanges and in the network of actors. The purpose of the first phase model is to mentally and practically prepare the patient for discharge on the day of surgery while the fourth phase model enables the early mobilisation of the patient and concludes with discharge on the day of surgery.

Similar in both models is the strong interdependency of the different caregivers, which requires a specific sequence of value exchanges. The network of actors in the care team consists of the following actors and includes both similarities and differences: anaesthetist (1), nurse specialist (1), pharmacist (1), radiologist (1), orthopaedic surgeon (1,4), patient (1,4), physiotherapist (1,4), doctor (4), nurse (4), the patient application (1,4). In phases 1 and 4, together with the other three phases, all presented actors recur once or more, except for the pharmacist.

In the models of both phase 1 and 4, the content of patient-involved value exchanges is dominated by bidirectional flows of information and questions. In addition, the transactions from caregivers often display caring characteristics such as advice, support, reassurance and motivation. The value attributes are also similar in both models. The caregivers derive a similar mix of values: quality, safety and insight. The patient mainly derives comfort in both models and insight in the first model.

## Discussion

In this study we investigated the organisational attributes of an outpatient THA care pathway. Our models depict the organisation of the outpatient THA care pathway in the critical phases of *diagnosis & preparation* and *recovery & discharge* to enable discharge on the day of surgery. The care pathway models visualise the network of actors and describe the corresponding value exchanges and their sequence.

We build on the existing THA clinical care pathway research by presenting the organisational aspects in a care pathway model design that is based on an in-depth investigation. Previous clinical studies have discussed the importance of the successful organisation of the care team [5] but without revealing details of how this was done. We fill this gap with a pathway model design of how to organise an outpatient THA care pathway, and we confirm the importance of the value exchange sequence for early discharge as discussed by Husted [6].

The care pathway model design is crucial to enable discharge on the day of surgery. We add to the clinical aspects of care provision the organisational aspects of actors connected by value exchanges. The organisation design impacts (1) a better education and communication of the patient, (2) the process quality of the care pathway and (3) higher efficiency of care. A triple benefit in addition to the clinical benefits.

This new THA pathway model supports healthcare professionals in the hospitals of THA to adopt the innovation. It transfers knowledge on how an outpatient THA care pathway can be organised to enable discharge on the day of surgery.

This knowledge assists hospitals in the integration of care [2] at an organisational level to foster alignment and collaboration across care departments with the purpose of enhancing quality, efficiency and patient satisfaction. To achieve full integration, the resources of the different care departments should be pooled with a view to creating a new organisation aimed at this specific group of patients, rather than trying to coordinate between existing organisational units [39].

We consider the care pathway models suitable for both regular and teaching hospitals because they share a similar organisation structure and have similar caregivers available. Hospitals can use care pathway models as a visual reference for healthcare professionals to compare the organisation of their THA care pathway to the presented models. This provides them with insights into the organisational differences that help to improve their care pathway.

Our contribution as designers is based on our ability of creating something new and envisioning a possible future care pathway rather than describing the present [40]. Moreover, we contribute our skill to visualise and produce a visual model that supports the communication of how the outpatient THA care pathway might be organised.

No models of any care pathway exist that link the network of actors by value exchanges. Models with a comparable structure have been found but differ in subject and level of analysis, such as business models [24] and care models [16].

### Limitations and further research

The case study methodology has proven to reveal in-depth insights into the object of analysis, yet there are limitations. In general, single case studies have limited external validity [28,29]. This limitation has been mitigated by drawing on multiple sources per finding and by checking the preliminary findings with another hospital. To test the transferability and provide support for applicability, the care pathway model has to be implemented in other (outpatient) THA care pathways. Based on thorough research, specific problems in the as-is outpatient THA care pathway at the case hospital were addressed in the care pathway models design. The design choices made in solving the problems and inefficiencies were partly derived from interviews with professional caregivers working in two different types of outpatient care pathways. Healthcare professionals from both hospitals stated that there would be no differences between their respective organisational networks for outpatient knee and hip care pathways. The differences only exist at the level of the value exchanges due to a focus on a different joint. This translates to differences in aspects such as patient information, anaesthesia and surgery.

The optimisations incorporated in the care pathway models have not been tested in reality and therefore future research is required to carry this out.

This paper focussed on the organisational characteristics of the outpatient THA care pathway. It should not be forgotten that the clinical characteristics play an equally large role in outpatient THA.

The modelling toolkit used for this research is of a visual nature, utilising symbols each accompanied by a descriptive word to map the value exchanges (Figure 1). Visual communication can be beneficial as a picture is worth a thousand words, but it also has its limits. Presenting symbols in isolation limits comprehensibility [41], as we also found. It may be beneficial to provide contextual definitions in advance of the interview to improve comprehensibility and cross-interview consistency. Nonetheless, any cards that were not comprehended by the interviewee were easily identified during his or her explanation of the value exchange. As an avenue for further research we suggest using visual modelling in the development of other pathways and exploring its usefulness and contribution to the communication of care pathways.

### Professional implications

In Figure 2 and 3 we presented the as-is care pathway model of the case hospital and the optimised design side by side. The differences between the care pathway models become apparent in a visual manner, and are the direct result of the optimisations that have been incorporated in the design.

For professionals involved in THA care pathways it is of interest to highlight their organisational differences with the outpatient THA care pathway models. A comparison of their care pathway model with the design will provide them with insight into how their care pathway is organised differently. This may inspire the caregivers to find out why these differences exist and may complement the diagnostic study in a bottom-up approach [42] to improve their integrated care pathway.

## Conclusion

This paper defines the concept of care pathway model design and presents two models of the most critical phases of the outpatient THA care pathway. The design addressed several existing problems and is an optimisation of the care pathway at the case hospital. The network of actors consists of the radiologist (1), anaesthetist (1), nurse specialist (1), pharmacist (1), orthopaedic surgeon (1,4), physiotherapist (1,4), nurse (4), doctor (4) and patient application (1,4). The critical value exchanges include patient preparation (mental and practical), patient education, aligned care team, efficient sequence of value exchanges, early patient mobilisation, flexible availability of the physiotherapist, functional discharge criteria, joint decision making and availability of the care team.

## Additional Files

The additional files for this article can be found as follows:

10.5334/ijic-2429.s1

DOI: https://doi.org/10.5334/ijic-2429.s1
