# Supplementary material for: Designing a Care Pathway Model – A Case Study of the Outpatient Total Hip Arthroplasty Care Pathway
Source: Int J Integr Care. 2017 Mar 9;17(1):2. doi: 10.5334/ijic.2429 (PMC5630075; doi:10.5334/ijic.2429)
Supplement: Supplementary file 1 [file ijic-17-1-2429-s1.pdf]

Appendix

Actor	Role
Anaesthetist	Examines the state of health of the patient to ensure that the right anaesthesia is administered, and provides support during surgery.
Doctor	Checks on the patient at the ward and discharges him or her. This role can be shared by an orthopaedic surgeon and another qualified doctor, depending on the availability of staff and a hospital's specific situation.
Informal caregiver	The informal caregivers support the patient along his or her journey.
Nurse	Supports the patients during their stay in the hospital. Is the main contact point for the patient and monitors his or her state of health. At discharge the nurse supports the patient in providing him or her with all relevant discharge documents.
Nurse specialist	Responsible for conducting the medical anamnesis and prescribing the medication.<br><br>A nurse specialist is trained to take over tasks of the doctor such as prescribing medication and conducting post-operative checks. Another name for such an actor is physician assistant.
Orthopaedic surgeon	Diagnoses, operates, checks and discharges the patient; he or she has final responsibility for the patient.
Patient	Travels along the outpatient THA care pathway.
Pharmacist	Checks which drugs the patient uses to ensure their availability when needed.
Physiotherapist	Teaches the patient to walk with crutches before admission, and supports the patient by mobilising him or her and guiding recovery after surgery.
Radiologist	Takes an x-ray of the patient's hip prior to the orthopaedic diagnosis.

Table 6 - Characteristics of the actors in the network of the care pathway model design
